# Comparative Analysis of Translucency in Different Thicknesses of Monolithic Zirconia Crowns

**DOI:** 10.1002/cre2.70132

**Published:** 2025-04-22

**Authors:** Azita Mazaheri, Ezatollah Jalalian, Arash Zarbakhsh, Mahshad Mazidabadi Farahani, Maryam Sayyari

**Affiliations:** ^1^ Department of Prosthodontics, Dental Branch Islamic Azad University Tehran Iran; ^2^ Tehran University of Medical Sciences Tehran Iran

**Keywords:** ceramics, color, dental materials, dental porcelain

## Abstract

**Objectives:**

Achieving optimal esthetics in dental restorations remains challenging, particularly with zirconia crowns, which, despite their durability, often exhibit less translucency than natural teeth. This study aimed to evaluate how varying thicknesses of monolithic zirconia crowns affect their translucency.

**Materials and Methods:**

In this experimental laboratory study, an initial model (a brass die) was scanned, and two dies, one black (9005 RAL), and one white (9010 RAL), were created from Resin according to the scanned file. Next, 30 zirconia crowns (ZrO_2_, Arum, High Translucent) were fabricated in three thicknesses: 0.5, 0.8, and 1.5 mm. The color and translucency of the samples were assessed under standard lighting conditions (D65) using a spectroradiometer, and the color parameters L*, b*, and a* were recorded. The translucency of each sample was measured using the CIEDE2000 and CIE‐Lab formulae. Data were analyzed using one‐way ANOVA and the Tamhane test.

**Results:**

Statistically significant changes in the color and translucency of the samples were observed with varying thicknesses (*p* < 0.001). Specifically, the translucency parameter (TP), L*, and b* values decreased as the thickness increased, whereas a* values remained relatively stable. The average TP values for CIE‐Lab ranged from 11.51 at 0.5 mm to 6.54 at 1.5 mm, and for CIEDE2000, they ranged from 8.19 at 0.5 mm to 4.82 at 1.5 mm.

**Conclusion:**

Within the limitations of this in vitro study, it can be concluded that reducing the thickness of monolithic zirconia restorations increases their translucency. Thinner zirconia restorations demonstrate a higher TP, which may offer improved esthetic integration in clinical applications.

## 1 Introduction

The primary objective of prosthetic treatment is to prevent further dental tissue loss while restoring esthetics and function with minimal biological impact. This can be challenging, as achieving both esthetics and the strength of restorative materials simultaneously is often difficult. Introduced into dentistry in the early 1990s, zirconia is now widely used as a core material for ceramics to enhance esthetics (Pjetursson et al. 2015). It has mechanical properties comparable to stainless steel and is among the strongest ceramics used in dentistry (Piconi and Maccauro 1999; Al‐Amleh et al. 2010). Beyond its strength, zirconia offers notable physical properties beneficial for dental restorations, including a color that closely resembles natural teeth and good opacity, reducing the need for masking or covering compared to metal bases (Manicone et al. 2007).

Zirconia crowns with veneering have notable disadvantages, including a high rate of veneer chipping, complications related to thermal expansion discrepancies, and the inert nature of zirconia, which impairs bonding with the veneer (Felberg et al. 2019). Advancements in digital technology have enhanced dental ceramics, leading to greater demand for durable, esthetic, and full ceramic restorations. CAD‐CAM technology allows for the use of various monolithic materials, such as zirconia and lithium disilicate, (Demir Sevinç et al. 2024; Kim et al. 2013) Monolithic ceramics are preferred due to their superior mechanical and esthetic properties, elimination of veneering porcelain, avoidance of chipping issues, and shorter clinical and laboratory processing times (Sarıyer and Subaşı 2024).

Factors like color, translucency, surface texture, and shape influence the esthetic quality of ceramic dental restorations. Despite its importance in providing a natural look, research has often overlooked translucency (Turgut et al. 2014). Translucency lies between full opacity and transparency, allowing light to pass through while scattering it, which softens the visibility of the underlying structure. It can be adjusted by managing light absorption, reflection, scattering, and transmission (Pérez et al. 2010; Tuncel et al. 2016). The most common measures of translucency are the translucency parameter (TP) and contrast ratio (CR), which quantify how a material's color varies against black‐and‐white backgrounds (Johnston et al. 1995).

Decreased translucency in dental ceramics has been observed as thickness increases. Most studies have focused on the translucency of dental ceramics up to 2 mm, typically the minimum thickness recommended by manufacturers (Kang et al. 2023). However, in clinical practice, restorations of varying thicknesses are required depending on the specific conditions of the teeth. Therefore, a precise understanding of the relationship between translucency and restoration thickness is crucial for enhancing the esthetic outcomes of restorations (Chaiyabutr et al. 2011). For feldspathic crowns and lithium disilicate crowns, it has been demonstrated that the color difference is influenced by restoration thickness. However, limited data are available on monolithic zirconia, necessitating further investigation into its optical properties and performance (Gomes et al. 2023; Xing et al. 2017; Bayindir and Koseoglu 2020; Alhgeg and Güven 2023). This study aimed to investigate the translucency of monolithic crowns at different thicknesses. It is hypothesized that the thickness of monolithic crowns has a significant effect on their translucency.

## 2 Materials and Methods

The sample size was calculated to be 10 in each group (total 30) based on the results of a similar study (Kim et al. 2016) using the one‐way analysis of variance (ANOVA) power analysis option in the PASS‐11 software, considering *β* = 0.02, *α* = 0.05, an average standard deviation of translucency of 4.3, and an effect size of 0.62.

A prepared tooth model was created for this study. The model featured a brass die with a height of 7 mm and a diameter of 5 m, including axial walls that converged at an angle of 10° and a chamfer finishing line with a depth of 0.8 mm. This prepared die was scanned using a Dental Scanner (DOF 3D, Korea). The scanned image was then transferred to a computer as an STL file and used to produce two resin dies (Detax, Germany) via a printer (Asiga MAX 3D, Australia.

Two dies were required to evaluate translucency: one black and one white. Thus, one die was colored black (9005 RAL) and the other white (9010 RAL) using acrylic varnish spray. Next, the crowns were anatomically designed to mimic a premolar tooth using Exocad software. All crown parts were designed with uniform thickness, except for a 4 mm^2^ area (2 × 2 mm) on the mid‐buccal side, which was designed with three different thicknesses: 0.5, 0.8, and 1.5 mm. An internal space of 35 μm was allocated for the acrylic varnish of the die.

Thirty monolithic zirconia crowns (ZrO_2_, Arum, Korea, High Translucent) were then fabricated using a milling machine (Arum, Korea), grouped into three sets of 10 (A, B, and C) according to the different thicknesses. The crowns were sintered in a MIHM‐VOGT dental furnace for 6 h at 1350°C. For porcelain glaze, all specimens underwent three repeated firings in a ceramic furnace (Koushafan Pars, KFP, Iran) using a glazing paste of CERABIEN ZR FC Paste (Kurarary, Italy). The temperature was increased to 950°C at a firing rate of 30°C/min, held for 30 s, and then cooled to 300°C at a rate of 15°C/min.

To measure the samples' translucency, the crowns were placed on the black die once and on the white die once. To negate the influence of background color, the black die was set against a standard black background, and the white die against a standard white background. The specifications for these backgrounds were black with L* = 7.60, a* = 0.45, and b* = 2.44, and white with L* = 88.83, a* = −4.95, and b* = −6.07. The samples were positioned 30 cm from the spectroradiometer.

The translucency of the samples was assessed under standard lighting conditions (D65) using a CS‐2000 Konica Minolta spectroradiometer. The measurement area was defined as a circular region with a diameter of 2.8 mm located at the center of the specimens. Color parameters L*, a*, and b* were measured and recorded. Each parameter was measured three times without moving the samples, and the average value was calculated. Measurements were conducted under consistent environmental conditions by a single operator, and the device was calibrated using a standard white color according to the manufacturer's instructions.

The color differences between the standard black and white backgrounds were calculated using CIE‐Lab and CIEDE2000 to evaluate translucency.

The formulas used were

Formula 1 (CIE‐Lab):

$$TP_{\mathrm{ab}}=\sqrt{{\left(L{*}_{B}-L{*}_{W}\right)}^{2}+{\left(a{*}_{B}-a{*}_{W}\right)}^{2}+{\left(b{*}_{B}-b{*}_{W}\right)}^{2}},$$

where *W* represents white and *B* represents black. L*_B_ is the L* value measured against the background color, while L*_W_ is the L* value measured against a white background.

Similarly, a*_B_ and a*_W_ are the * values for the background and white backgrounds, respectively, and b*_B_ and b*_W_ are the b* values.

The CIEDE2000 (1:1:1) color difference formula was also used to calculate the translucency parameter (TP_00_).

Formula 2:

$$\Delta E^{0}=\sqrt{{\left(\frac{\Delta L^{\prime }}{\left(K_{L}S_{L}\right)}\right)}^{2}+{\left(\frac{\Delta C^{\prime }}{\left(K_{C}S_{C}\right)}\right)}^{2}+{\left(\frac{\Delta H^{\prime }}{\left(K_{H}S_{H}\right)}\right)}^{2}+R_{t}\cdot \;{\left(\frac{\Delta C^{\prime }}{\left(K_{C}S_{C}\right)}\right)}^{2}\cdot \;{\left(\frac{\Delta H^{\prime }}{\left(K_{H}S_{H}\right)}\right)}^{2}.}$$

In this formula, *C* denotes chroma, and *H* denotes hue in both the white and black backgrounds. The weighting functions *S_L_*, *S_C_*, and *S_H_* adjust for color differences in areas where the samples are placed against white and black backgrounds. The parameters *K_L_*, *K_C_*, and *K_H_* are laboratory condition modifiers set to 1 for this study.

Several variables were analyzed statistically in this study, including the L*, a*, b*, ΔL*, Δa*, and Δb* parameters, as well as the TP calculated using the CIE‐Lab and CIEDE2000 formulas. One‐way ANOVA was applied to evaluate the statistical significance of translucency differences across the three sample thicknesses (0.5, 0.8, and 1.5 mm) for each translucency measurement method (CIE‐Lab and CIEDE2000). Post‐hoc analysis using the Tamhane test was performed to identify specific group differences. The significance level was set at *p* < 0.05.

## 3 Results

The L*, a*, and b* values for sample thicknesses of 0.5, 0.8, and 1.5 mm in different backgrounds are presented in Table 1.

**Table 1:** Average color coordinates (L*, a*, and b*) for samples of different thicknesses by background color.

Thickness (mm)	Background	Parameter	Mean (standard deviation)	Minimum	Maximum
0.5	White	L*	88.6 (0.7)	87.4	89.9
		a*	−1.2 (0.6)	−2.7	−0.6
		b*	3.3 (1.5)	1.9	6.6
	Black	L*	77.9 (1.0)	76.6	79.0
		a*	−1.2 (0.5)	−2.4	−0.8
		b*	−0.9 (1.1)	−2.0	1.5
0.8	White	L*	87.6 (0.6)	86.6	88.3
		a*	−0.9 (0.1)	−1.1	−0.6
		b*	1.9 (0.3)	1.3	2.3
	Black	L*	78.9 (0.5)	78.2	79.8
		a*	−1.0 (0.1)	−1.2	−0.8
		b*	−1.1 (2.1)	−2.3	4.8
1.5	White	L*	87.4 (0.6)	86.5	88.5
		a*	−1.2 (0.2)	−1.5	−0.9
		b*	1.3 (1.1)	−1.6	2.5
	Black	L*	81.6 (0.7)	80.8	82.9
		a*	−1.3 (0.2)	−1.6	−1.1
		b*	−1.2 (1.1)	−2.2	1.6

Table 2 indicates the ΔL*, Δa*, and Δb* parameters categorized by sample thickness.

**Table 2:** Average color coordinates ΔL*, Δa*, and Δb* for samples by thickness.

Thickness	Parameter	Mean (standard deviation)	Minimum	Maximum
0.5 mm	ΔL*	10.7 (1.3)	8.5	12.5
	Δa*	0.1 (0.1)	0.0	0.3
	Δb*	4.2 (0.5)	3.3	5.1
0.8 mm	ΔL*	8.7 (0.6)	7.9	9.8
	Δa*	0.1 (0.0)	0.0	0.2
	Δb*	3.5 (0.4)	2.7	3.9
1.5 mm	ΔL*	5.8 (0.7)	4.2	6.7
	Δa*	0.1 (0.0)	0.0	0.2
	Δb*	3.0 (0.3)	2.7	3.8

The average values of TP using CIE‐Lab for samples with thicknesses of 0.5, 0.8, and 1.5 millimeters were 11.5119, 9.4698, and 6.5430, respectively (*p* < 0.001). The average TP CIEDE2000 values for thicknesses of 0.5, 0.8, and 1.5 mm were 8.1826, 6.8272, and 4.8233, respectively (*p* < 0.001). (Table 3).

**Table 3:** Comparison of translucency by thickness and calculation method (CIEDE2000, CIE‐Lab).

Formula	Thickness	Mean TP (standard deviation)	Minimum	Maximum	*p*‐value
CIE‐Lab	0.5 mm	8.2 (0.9)	6.5	9.4	<0.001
	0.8 mm	6.8 (0.4)	6.3	7.6	
	1.5 mm	4.8 (0.3)	4.4	5.3	
CIEDE2000	0.5 mm	11.5 (1.3)	9.1	13.3	<0.001
	0.8 mm	9.5 (0.6)	8.8	10.6	
	1.5 mm	6.5 (0.5)	5.6	7.4	

Abbreviation: TP, translucency parameter.

Table 4 presents TP differences between sample thicknesses and compares them to perceptibility and acceptability thresholds. All thickness comparisons (0.5–0.8 mm, 0.8–1.5 mm, and 0.5–1.5 mm) exceed the perceptibility threshold for both TPs. Regarding the acceptability threshold, the 0.8–1.5 mm and 0.5–1.5 mm thickness differences exceed the threshold for ΔTP_00_.

**Table 4:** Translucency parameter differences between sample thicknesses.

Thickness comparison	CIEDE2000 difference	CIE‐Lab difference	Perceptibility threshold (ΔTP_00_)	Acceptability threshold (ΔTP_00_)	Perceptibility threshold (ΔTPab)	Acceptability threshold (ΔTP_ab_)
0.5–0.8	2.0	1.4	0.6	2.6	1.3	4.4
0.8–1.5	2.9	2.0				
0.5–1.5	5.0	3.4				

## 4 Discussion

The optical properties of ceramic restorations are crucial in clinical practice as patients increasingly seek functional efficacy and esthetic integration with their natural dentition, particularly in esthetic areas (Miyazaki et al. 2009). The translucency of ceramic restorations, which can range from allowing light transmission to completely covering the underlying tooth structure, is significantly influenced by the type and thickness of the ceramic material. These parameters play a critical role in determining both restorative coverage and translucency (Wang et al. 2013; Subaşı et al. 2018; Arif et al. 2019). Monolithic zirconia is known for its high flexural strength, natural tooth‐like appearance, minimal wear on antagonist teeth, and reduced need for tooth preparation (Stober et al. 2014). Therefore, this study aimed to evaluate the translucency of monolithic zirconia crowns at different thicknesses. The results of the study indicate a statistically significant difference in the color and translucency of the samples with changes in thickness (*p* < 0.001). Specifically, TP, L*, and b* values decreased with increasing thickness, while a values did not significantly change. Thus, the initial null hypothesis was confirmed.

A precise understanding of the relationship between translucency and restoration thickness is crucial for optimizing the esthetic outcomes of dental restorations (Chaiyabutr et al. 2011). The recommended thickness range for translucent zirconia is 0.5–2.0 mm (Bayindir and Koseoglu 2020; Tabatabaian et al. 2020, 2021). This study examined the effect of 0.5‐, 0.8‐, and 1.5‐mm thicknesses on the translucency of monolithic zirconia restorations.

Various studies use spectrophotometers, colorimeters, and spectroradiometers for color measurement (Alghazali et al. 2011; Tabatabaei et al. 2019; Khorshidi et al. 2024). Spectrophotometers and spectroradiometers offer greater adaptability and can detect metamerism, unlike colorimeters. While most translucency studies use spectrophotometers, they may produce systematic errors due to light scattering at sample edges. Spectroradiometers are increasingly used in dentistry for more accurate measurements (Li et al. 2009; Lim et al. 2010). This study employed the Spectroradiometer CS‐2000‐Konica Minolta to assess optical parameters.

The optical properties of teeth and dental restorations are assessed using translucency, hue, value, and chroma (Kelly et al. 1996). The CIE‐Lab color system is commonly employed in dentistry to evaluate these attributes (Johnston et al. 1995). In this system, L* denotes lightness (ranging from 0 for black to 100 for white), a* indicates the green‐red spectrum (negative values for green, positive for red), and b* represents the blue‐yellow spectrum (negative values for blue, positive for yellow) (Lim et al. 2010). Research indicates that changes in L* are more critical for detecting color differences in restorations than variations in a* and b*, as the human eye is more sensitive to lightness variations than to color differences (Marcucci 2003).

The study found that Δb* and ΔL* values decreased significantly with increasing thickness across 0.5, 0.8, and 1.5 mm thicknesses. These results are consistent with findings from Kim et al. (2016), Saker and Özcan (2021), Bayindir and Koseoglu (2020), and Kang et al. (2023). However, Δa* showed inconsistent trends: it decreased from 0.5 to 0.8 mm but increased from 0.8 to 1.5 mm. This variability aligns with studies by Bayindir and Koseoglu (2020) and Tabatabaian et al. (2020), which suggest that changes in Δa* are less critical than variations in ΔL* and Δb* across different thicknesses.

TP is a crucial factor in assessing the optical properties of restorations. Research indicates that color perception is affected by factors such as sample thickness and surface texture. *ΔE* thresholds for perceptibility by the human eye range from 1.0 to 3.7, with acceptable ranges varying between 1.7 and 6.8. Since previous research has shown that the CIEDE2000 formula is more clinically relevant than CIE‐Lab, this study also assessed translucency using CIEDE2000 (Paravina et al. 2019). For dental materials, the perceptibility threshold is ΔTP_00_ = 0.6 and the acceptability threshold is ΔTP_00_ = 2.6, while the perceptibility threshold for ΔTP_ab_ is 1.3 and the acceptability threshold is 4.4 (Salas et al. 2018).

Values below TP 2.0 are considered opaque because they obscure a black background (Chaiyabutr et al. 2011). In this study, all samples, including those with a maximum thickness of 1.5 mm, exhibited translucency values significantly above the perceptibility and acceptability thresholds for both ΔTP_00_ and ΔTP_ab_. This confirms that all samples allowed sufficient light transmission and background visibility, aligning with the findings from Baldissara et al. (2018).

Consistent with the studies by Kim et al. (2016), Sulaiman et al. (2015), Church et al. (2016), and Malkondu et al. (2016), the data revealed that translucency, as measured by CIE‐Lab, decreases with increasing thickness. This reduction in translucency was statistically significant across all sample groups.

The results indicated that TP for all sample thicknesses exceeded the perceptibility thresholds for both ΔTP_00_ and ΔTP_ab_, confirming that changes in translucency are detectable by the human eye. This aligns with the findings from Pecho et al. (2015).

Consistent with the CIE‐Lab results, the CIEDE2000 data also showed a decrease in translucency with increased sample thickness, confirming the results of Tabatabaian et al. (2020).

Compared to human dentin and enamel, which have translucency values of 16.4 and 18.7 at 1.0 mm thickness (Yu et al. 2009), the samples in this study had significantly lower translucency values. These results indicate a need for further improvement in the optical properties of these materials (Conrad et al. 2007).

This study successfully highlights the impact of thickness on the translucency of high translucent zirconia restorations, providing valuable data and offering practical benchmarks for dental materials. However, the study's limitations include its in vitro nature, which may not fully capture clinical conditions, and the absence of cementation, which could influence translucency results. Additionally, the research focused on a single shade (A1) and high translucency samples, which may not represent the full spectrum of clinical scenarios. Future research should address these limitations by conducting in vivo studies, including cementation in sample evaluations, and expanding the range of shades and translucency levels to provide a more comprehensive understanding of how these factors affect translucency and color in clinical practice.

## 5 Conclusion

Within the limitations of this in‐vitro study, it can be concluded that reducing the thickness of monolithic zirconia restorations enhances their translucency. Thinner zirconia restorations exhibit a higher TP, which could suggest a potential for improved esthetic integration in clinical applications.

## Author Contributions

**Azita Mazaheri:** conception and design of the study, methodology, final approval of the manuscript. **Ezatollah Jalalian:** resources and data curation, final approval of the manuscript. **Arash Zarbakhsh:** supervision, project administration, funding acquisition, final approval of the study. **Mahshad Farahani Zeidabadi:** acquisition of the data, data analysis, original draft preparation, final approval of the manuscript. **Maryam Sayyari:** critical revision, drafting the article, final approval of the manuscript.

## Ethics Statement

The School of Dentistry, Islamic Azad University, Tehran's ethics committee approved the study.

## Consent

The authors have nothing to report.

## Conflicts of Interest

The authors declare no conflicts of interest.

## Data Availability

The data that support the findings of this study are available from the corresponding author upon reasonable request.
